# Identification of SNPs associated with variola virus virulence

**DOI:** 10.1186/1756-0381-6-3

**Published:** 2013-02-14

**Authors:** Anne Gatewood Hoen, Shea N Gardner, Jason H Moore

**Affiliations:** 1 Department of Community and Family Medicine, The Geisel School of Medicine at Dartmouth, Dartmouth College, One Medical Center Drive, Lebanon, NH 03756, USA; 2 Computations/Global Security, Lawrence Livermore National Laboratory, P.O. Box 808, L-174, Livermore, CA, 94551, USA; 3 Department of Genetics, Institute for Quantitative Biomedical Sciences, The Geisel School of Medicine at Dartmouth, Dartmouth College, One Medical Center Drive, Lebanon, NH 03756, USA

**Keywords:** Smallpox, Variola virus, Single nucleotide polymorphisms, Multifactor dimensionality reduction

## Abstract

**Background:**

Decades after the eradication of smallpox, its etiological agent, variola virus (VARV), remains a threat as a potential bioweapon. Outbreaks of smallpox around the time of the global eradication effort exhibited variable case fatality rates (CFRs), likely attributable in part to complex viral genetic determinants of smallpox virulence. We aimed to identify genome-wide single nucleotide polymorphisms associated with CFR. We evaluated unadjusted and outbreak geographic location-adjusted models of single SNPs and two- and three-way interactions between SNPs.

**Findings:**

Using the data mining approach multifactor dimensionality reduction (MDR), we identified five VARV SNPs in models significantly associated with CFR. The top performing unadjusted model and adjusted models both revealed the same two-way gene-gene interaction. We discuss the biological plausibility of the influence of the SNPs identified these and other significant models on the strain-specific virulence of VARV.

**Conclusions:**

We have identified genetic loci in the VARV genome that are statistically associated with VARV virulence as measured by CFR. While our ability to infer a causal relationship between the specific SNPs identified in our analysis and VARV virulence is limited, our results suggest that smallpox severity is in part associated with VARV strain variation and that VARV virulence may be determined by multiple genetic loci. This study represents the first application of MDR to the identification of pathogen gene-gene interactions for predicting infectious disease outbreak severity.

## Findings

Smallpox, the only human infectious disease to have been eradicated, remains a threat as a potential agent of bioterrorism. Mortality rates during natural outbreaks of smallpox varied widely, a feature partially attributed to strain-specific virulence of the etiological agent, the orthopoxvirus variola virus (VARV) [1]. An understanding of the genetic determinants of virulence of VARV is critical for predicting the potential of different strains for causing severe epidemics.

We analyzed the genome-wide single nucleotide polymorphisms (SNPs) of a collection of 35 temporally, geographically and epidemiologically diverse VARV isolates housed at the US Centers for Disease Control and Prevention’s secure repository (previously described in [2]) for associations with VARV virulence. We investigated the only known metric of innate virulence of these isolates, their case fatality rates (CFRs), which range from <1-30% [2]. Previously, outbreaks of smallpox have been classified as ‘major’ when they exhibit greater than 10 percent CFRs. Because CFR can be considered a complex phenotype of VARV that is likely mediated by, among other factors, multiple viral genetic loci, we focused on identifying combinations of SNPs that may interact in their associations with CFR. We used the non-parametric data mining approach known as multifactor dimensionality reduction (MDR), described previously [3]. Briefly, MDR evaluates candidate models based on all possible combinations of a given number of SNPs and applies a naive Bayes classifier with cross-validation to estimate the testing accuracy of each candidate model. The statistical significance of the model that maximizes the testing accuracy is then assessed by permutation testing. MDR has been used extensively to detect epistatic interactions in genome-wide association studies of complex human diseases such as cancer, autism and cardiovascular disease. This study is novel in its application of MDR to pathogen genome data.

From the full-genome sequences of the 35 isolates we studied, we identified 1730 SNPs using kSNP with k=25 [4]. We built a list of candidate SNPs for MDR analysis by removing SNPs unlikely to have relevance to VARV virulence, including 979 synonymous SNPs and another 192 SNPs located in hypothetical proteins. Of the remaining 559 SNP loci, 126 were missing character states for one or more isolates. We eliminated from our analysis 92 SNPs that were missing data for more than one isolate. The remaining missing character states were imputed by replacing them with the most commonly observed character state for that SNP. 345 SNPs with allele frequencies of less than 10 percent (in fewer than 4 genomes) were also removed from the dataset, leaving 122 candidate SNPs. We grouped SNP loci that were perfectly collinear, that is, were in complete linkage disequilibrium in our sample of isolates. These loci are redundant and impossible to distinguish in our models. This left us with 21 individual SNPs or SNP groups as candidate attributes for our MDR models.

Our outcome of interest was CFR, coded as a binary variable where 0 represented CFRs ≤ 10 percent and 1 represented CFRs > 10 percent. Of the 35 isolates, 18 were associated with CFRs > 10 percent. Preliminary analyses revealed that CFR is significantly associated with geographic location, namely, whether the outbreak occurred on the African continent (Fisher’s exact test, *p*<0.001); therefore, in adjusted models we included a binary covariate where 0 represented strains isolated from non-African outbreaks and 1 represented strains isolated from African outbreaks. We fit one-way, two-way and three-way SNP-SNP interaction models using MDR. All models were fit using five-fold cross-validation and evaluated for significance using 1000 random permutations [5].

Previous work has shown that these isolates form two major phylogenetic clades, with the clade containing Asian and non-West African VARV variants, referred to as primary clade I in [6], exhibiting different biological properties than the clade containing West African and South American variants [6,7]. Accordingly, we investigated the sensitivity of our modeling approach to phylogenetic differences by performing the same analysis on the set of isolates belonging to primary clade I. This model could not be adjusted for a geographic association between CFR and African origin because CFR and geographic origin are perfectly correlated among isolates in this clade.

Our models identified 5 individual SNPs and 2 groups of perfectly collinear SNPs that significantly predict smallpox CFR (Table 1). SNPs are hereafter referred to by their positions in the genome of the reference strain gi|94484460|gb|DQ441420.1| Variola virus strain Bangladesh 1974 (nur islam). These SNPs represent loci in 19 different VARV genes (Table 2). We used testing balanced accuracy, a measure of model accuracy that avoids inflating performance estimates due to unbalanced data to measure the performance of each model. The same two-way SNP-SNP interaction was identified in both the top-performing unadjusted and adjusted models (Table 1). The top-performing unadjusted model slightly outperformed the corresponding adjusted model; however, the difference in model testing balanced accuracy was small and both models were significant. When we restricted our analysis to only the isolates in primary clade I, we identified two models with equal testing balanced accuracy values, a two-way model and a three-way model. However, the two-way model performed better was more consistently selected in cross-validation. In all three analyses, the top-performing model revealed the same two-way effect of an interaction between SNP 127469 and one or more SNPs in group 1 on CFR.

**Table 1:** Summary of models evaluated by MDR for predicting smallpox outbreak CFRs using VARV SNPs

	**All isolates**								Primary cladel I* isolates only			
**No. SNPs consi-dered**	**Unadjusted models**				**Adjusted model**				**Unadjusted models**			
	**SNPs in best model**	**CVC**	**Testing balance**	**P**	**SNPs in best model**	**CVC**	**Testing balance**	**P**	**SNPs in best model**	**CVC**	**Testing balance**	**P**
1	18083	3/5	0.8268	0.018	127469	5/5	0.7365	0.020	183426	4/5	0.8333	0.010
2	Group 1,127469	3/5	0.8562	0.003	Group 1, 127469	4/5	0.8227	<0.001	Group 1, 127469	4/5	0.9042	<0.001
3	75336, 127469, 183083	3/5	0.8268	0.018	127469, Group 2, 42293	3/5	0.8067	<0.001	Group 1, Group 2, 127469	3/5	0.9042	<0.001

*From reference [6].

CVC: cross valid consistency.

**Table 2:** SNPs identified in MDR analysis of associations between VARV genetic variation and smallpox outbreak CFRs

**Group**	**SNP**	**Nucleotide variant (allele in reference strain/alternative allele)**	**GenBank gene accession number**	**Amino acid in reference strain/alternative allele**	**Amino acid position in protein**	**Protein name**	**Biological function**
N/A	183083	C/T	ABG43367	H/Y	160	Crm-B secreted TNF-alpha-receptor-like protein	Host defense modulator
1	28347	A/G	ABG43202	E/K	296	Ser\Thr kinase	Phosphorylates virion proteins involved in assembly
	126854	T/C	ABG43300	D/N	12	Cowpox A-type inclusion protein	Unknown
	136573	A/G	ABG43313	D/N	35	IEV transmembrane phosphoprotein	Interacts with A33R and needed for actin tail formation
	151088	T/C	ABG43336	T/I	22	Hemagglutinin protein	Type-I membrane glycoprotein, inhibits cell fusion
N/A	127469	A/G	ABG43301	S/L	52	Cowpox A-type inclusion protein	Unknown
N/A	75336	T/C	ABG43252	Y/H	183	DNA-dependent RNA polymerase subunit rpo147	Viral transcription
2	6738	G/T	ABG43172	D/A	169	Ankyrin-like protein	Unknown
	7008	T/C	ABG43172	K/R	79	Ankyrin-like protein	Unknown
	8300	T/C	ABG43175	D/N	111	Ankyrin-like protein	Unknown
	8333	G/T	ABG43175	N/H	100	Ankyrin-like protein	Unknown
	20739	A/G	ABG43191	Q/*	88	Interferon resistance protein	Host defense modulator
	24914	T/C	ABG43196	G/S	151	Ribonucleotide reductase small subunit	Deoxyribonucleoside diphosphate metabolism
	74679	A/G	ABG43251	T/I	2	IMV membrane protein	Involved in viral-cell entry and virus-infected cell-cell fusion
	127554	G/A	ABG43301	C/R	22	Cowpox A-type inclusion protein	Unknown
	143136	T/C	ABG43324	H/R	197	Hydroxysteroid dehydrogenase	Steroid biosynthesis
	147901	A/G	ABG43331	D/N	194	DNA ligase	DNA ligation during DNA repair; DNA recombination; DNA replication
	150364	T/C	ABG43334	S/L	57	Kelch-like ring canal protein	Unknown
	162026*	T/C	ABG43349	Q/*	105	Ser/Thr kinase-like protein	Unknown
			ABG43350	F/F	4	SPI-2/CrmA IL-1 convertase	Inhibits Fas-mediated apoptosis (host defense modulator)
N/A	42293	G/A	ABG43215	E/G	147	IMV protein	Unknown
N/A	183426	A/C	ABG43367	A/E	276	Crm-B secreted TNF-alpha-receptor-like protein	Host defense modulator

IEV: intracellular enveloped virion; IMV: intracellular mature virion.

*SNP is in two overlapping open reading frames.

SNP 127469 was involved in all models with the exception of the one-way unadjusted models. This SNP falls within the gene encoding the cowpox A-type inclusion protein. In some orthopoxviruses, the A-type inclusion protein forms inclusion bodies in the cytoplasm of infected cells, into which mature virus particles are embedded. The presence of inclusion bodies probably enhances survival and dissemination in the environment [8,9]. VARV, like other orthopoxviruses, has a conserved A-type inclusion protein gene; however, VARV-infected cells accumulate large quantities of the protein without forming typical inclusion bodies, which suggests that it may have some other function. The A-type inclusion protein has been shown in the prototypical orthopoxvirus vaccinia virus to be highly immunogenic, and variants of vaccinia with mutations in the A-type inclusion protein gene exhibit reduced immunogenicity [10].

The other variable in our top models, group 1, represents a group of 4 perfectly collinear SNPs that cannot be distinguished among the strains in our sample. SNP 28347 falls within a gene for a serine/threonine kinase that phosphorylates virion proteins involved in assembly. SNP 126854 falls within a second gene for the cowpox A-type inclusion protein. SNP 136573 is a polymorphism in the gene encoding A36R, a transmembrane phosphoprotein of the intracellular enveloped virion (IEV) form. The IEV is the precursor to the extracellular cell-associated enveloped virion, which requires this protein for producing the actin tails that push it away from the cell surface and allow it to disseminate to adjacent cells. Loss of A36R results in a small plaque phenotype *in vitro* and in reduced virulence *in vivo*[11]. SNP 151088 is a locus in the gene for the hemagglutinin protein. It is found in the extracellular enveloped virus form of VARV. In the vaccinia virus model, in which it is known as the A56 protein, it is able to bind two viral proteins, K2, a serine protease inhibitor, and VCP, the vaccinia virus complement control protein, to the surface of infected host cells. With A56, K2 reduces the amount of virus superinfecting an infected cell and prevents the fusion of infected cells, while the VCP complex inhibits complement activity. Deletion of this gene impacts vaccinia virus virulence [12].

Other models identified by our MDR analysis performed inferiorly to these; however, they allowed us to identify SNPs that were significantly associated with CFR. SNP 183083 was found in two relatively high-performing models, both the one-way and three-way unadjusted models. This SNP is in the gene for the CrmB secreted TNF-alpha-receptor-like protein. This is a TNF-alpha receptor expressed by poxviruses that blocks cytokine activity to interfere with the host immune function, thereby enhancing viral virulence [13]. Additional SNPs identified in our analysis are shown in Table 2 and include loci in genes that encode additional host defense modulators and proteins critical to the viral life cycle.

This study represents a novel application of MDR to studying pathogen genome-wide associations with infectious disease outbreak severity. Of the models we evaluated using MDR, the best-performing model predicted smallpox outbreak CFR with a two-way VARV gene-gene interaction. While we are limited in our ability to infer a causal relationship between the interacting SNPs identified in our analysis and smallpox outbreak severity, the genes implicated have potential biological plausibility as determinants of VARV virulence and may represent targets for future laboratory-based investigations. The results of this study reinforce existing evidence that smallpox outbreak severity may be in part a consequence of the VARV strain associated with the outbreak and suggests that VARV virulence is a complex trait influenced by multiple genetic loci.

## Abbreviations

CFR: Case fatality rate; EEV: Extracellular enveloped virus; IEV: Intracellular enveloped virion; MDR: Multifactor dimensionality reduction; SNP: Single nucleotide polymorphism; VARV: Variola virus.

## Competing interests

The authors declare that they have no competing interests.

## Authors’ contributions

AGH participated in the study design, performed the statistical analysis and drafted the manuscript. SNG performed SNP analysis and contributed to the interpretation of the data and the drafting of the manuscript. JHM conceived of the study, participated in the study design and statistical analyses and contributed to interpretation of the data and the drafting of the manuscript. All authors read and approved the final manuscript.
